# Metabolic Reprogramming and Vulnerabilities in Cancer

**DOI:** 10.3390/cancers12010090

**Published:** 2019-12-30

**Authors:** Costas A. Lyssiotis, Deepak Nagrath

**Affiliations:** 1 Department of Molecular and Integrative Physiology, University of Michigan, Ann Arbor, MI 48109, USA; 2 Division of Gastroenterology and Hepatology, Department of Internal Medicine, University of Michigan, Ann Arbor, MI 48109, USA; 3 Rogel Cancer Center, University of Michigan, Ann Arbor, MI 48109, USA; 4 Department of Biomedical Engineering, University of Michigan, Ann Arbor, MI 48109, USA; 5 Department of Chemical Engineering, University of Michigan, Ann Arbor, MI 48109, USA; 6 Biointerfaces Institute, University of Michigan, Ann Arbor, MI 48109, USA

**Keywords:** cancer metabolism, tumor microenvironment, cancer associated fibroblasts, reactive oxygen species, redox, amino acids, carbohydrates, lipids, nucleotides, iron

## Abstract

Metabolic programs are rewired in tumors to support growth, progression, and immune evasion. A wealth of work in the past decade has delineated how these metabolic rearrangements are facilitated by signaling pathways downstream of oncogene activation and tumor suppressor loss. More recently, this field has expanded to include metabolic interactions among the diverse cell types that exist within a tumor and how this impacts the immune system. In this special issue, 17 review articles discuss these phenomena, and, alongside four original research manuscripts, the vulnerabilities associated with deregulated metabolic programming are highlighted and examined.

The reprogramming of cellular metabolism is a hallmark feature observed across cancers [1]. Contemporary research in this area has led to the discovery of tumor-specific metabolic mechanisms and illustrated ways that these can serve as selective, exploitable vulnerabilities. In this Editorial, we provide a high-level overview of the central themes from among the 21 review articles and original research studies in the Special Issue on *Metabolic Reprogramming and Vulnerabilities in Cancer*. 

Nutrient acquisition and cancer growth: Metabolic programs are rewired in cancer cells to facilitate macromolecular biosynthesis required for cellular proliferation and tumor growth. These programs are frequently driven by oncogenic signaling pathways. This observation gave rise to the notion that metabolic pathways may exist that cancer cells are over-reliant or uniquely dependent upon and could therefore serve as drug targets. Accordingly, there has been considerable interest in mapping the regulation and activity of metabolic pathways across virtually every type of cancer. In this Special Issue, a detailed review on the regulation of glucose metabolism in pancreatic cancer is provided by Yan et al. [2], nucleotide metabolism by Villa et al. [3], and amino acid metabolism by Choi and Coloff [4]. 

In order to fuel such biosynthetic pathways, cancer cells employ a variety of mechanisms to enhance the uptake and utilization of nutrients including the over-expression of carbohydrate, amino acid, and lipid transporters as well as the activation of other bulk nutrient uptake programs (Figure 1A). Based on their abundance in circulation and the ubiquity of metabolic pathways into which they can integrate, glucose and glutamine are two of the primary nutrient inputs that support the growth of cancer cells. However, much of the work on these important fuels has been determined using cell culture models, where nutrient and oxygen concentrations, matrix effects, inter-cellular interactions, among other factors, do not accurately reflect the physiochemical makeup of a tumor. Accordingly, the relevance of in vitro described glucose and glutamine pathways in tumors in vivo is now being delineated. Here, several reviews tackle this challenging topic as it relates to glutamine [5,6,7]. 

In the research article from Guda et al., the authors illustrate that glucose uptake correlates with aggressive features of brain cancer and describe new strategies to target this axis [8]. Outside of the brain, access to glucose for some tumors can be more restricted, and alternate pathways must be employed to support growth in its absence. For example, in the research article by Hodakoski et al., the authors found that non-small cell lung cancers employ macropinocytosis, a process of bulk extracellular engulfment or “cell drinking” to obtain nutrients to support glucose independence [9]. Notably, Hodakoski et al. found that protein-derived alanine obtained via macropinocytosis and released upon lysosomal protein breakdown served as a gluconeogenic intermediate.

Stress resistance: Metabolic pathways are also reprogrammed in cancer cells to enable resistance to intrinsic stressors including oxidative stress and apoptosis as well as to promote resistance to therapies. Reactive oxygen species (ROS) are byproducts of metabolism that can activate signaling pathways or damage biomolecules including DNA. In cancer cells, the production of ROS are often elevated as a consequence of metabolic rearrangements and are selected to promote genetic mutations (Figure 1B). Petronek et al. provide an up-to-date review on the role of iron as a central player in these processes [10]. However, if unchecked, excessive ROS can also be toxic to cancer cells. As such, cancer cells simultaneously drive antioxidant pathways that quench ROS. These are detailed from a metabolic perspective in the reviews by Purohit et al. [11] and Cockfield and Schafer [12]. Similarly, the role of cysteine in mitigating ROS is reviewed by Combs and DeNicola [13]. 

The Nrf2 transcription factor responds to oxidative stress by activating an antioxidant signaling program. In a research article by Haley et al., the authors found that inhibition of Nrf2 promoted epithelial to mesenchymal transition (EMT) of non-small cell lung cancer cells and facilitated metastasis [14]. These studies provide new insights into the role of this important antioxidant signaling program in cancer cell dissemination. Finally, cancer cells must also protect themselves against cell death programs. Sharma et al. present an argument for the rewiring of metabolism as an active player that directly maintains survival and defends against apoptosis [15].

Metabolism in the tumor microenvironment: Tumors are composed of a complex myriad of malignant and non-malignant cells that cooperate to support the growth of the tumor and the evasion of the anti-tumor immune system [16]. This collective group of cells is known as the tumor microenvironment (TME), and can play deterministic roles directing the dysregulated metabolic traits of the cancer cells (Figure 1C). For example, Loponte et al. discuss how differential nutrient access, cell–cell interactions, and intrinsic genetic programs lead to metabolic heterogeneity among different malignant cells in a tumor [17]. These sorts of interactions also impact the redox state, for example, based on the local oxygenation state, and Weinberg et al. discuss how this impacts metabolic programs in malignant cells [18]. 

The TME can also reprogram the metabolism of non-tumorigenic cells within solid tumors, which supports their survival and growth and that of the tumor. For example, a major component of many tumors is the activated stroma comprised of cancer associated fibroblasts (CAFs). These cells have been reported to play an ever increasing role in tumor metabolism, as detailed in the review by Sanford-Crane et al. [19]. Finally, outside of the TME, Ramteke et al. describe how systemic factors including blood glucose levels, circulating insulin, growth factors, and other nutrients impacts tumor growth [20].

Therapeutic targeting of metabolic vulnerabilities: The detailing of metabolic programs in cancer cells and the TME has suggested numerous vulnerabilities and opened the door to new drug targets and therapeutic options [21]. This topic takes center stage across the reviews and research articles in this Special Issue. In this vein, it is important to remember that metabolism-targeted therapies are among the first and most successful chemotherapeutic paradigms [22]. As a prime example, Naffouje et al. detail the storied history of inosine monophosphate dehydrogenase (IMPDH) inhibitors, an enzyme that acts at a central node in nucleotide biosynthesis [23]. Deregulated metabolic programs also promote resistance to many established therapies [24]. In the research work by Luanpitpong et al., the authors illustrate that upregulation of lipid import and the formation of lipid droplets promotes resistance to the proteasome inhibitor bortezomib in mantle cell lymphoma [25].

New insights into metabolism have also been leveraged to expand current modalities or design new drug targets. For example, Zhou and Wahl provide a contemporary perspective on the deregulated metabolic state in brain tumors, detailing insights into how these vulnerabilities can sensitize tumor cells to radiation and epigenetic therapies [26]. Of note, the authors describe how the oncometabolite 2-hydroxyglutarate (2HG), a product of the mutant isocitrate dehydrogenase 1 (IDH1) enzyme, rewires the epigenome, creates a metabolic vulnerability, and sensitizes these tumors to DNA damage-targeted combination therapy. The discovery of mutant IDH1, and the function of the oncometabolite 2HG, has paved the way for one of the most successful metabolism-targeted modalities in oncology in the modern era [27].

Knowledge about the metabolic programs operative in cancer has grown tremendously in the past 15 years. Many of the emerging themes in this rapidly growing field are comprehensively detailed in this collection of up-to-date reviews and research articles. They also contain insightful perspectives on the emerging areas of study and the associated therapeutic opportunities. It is our hope that readers find them to be timely and informative.

## Figures and Tables

**Figure 1:**
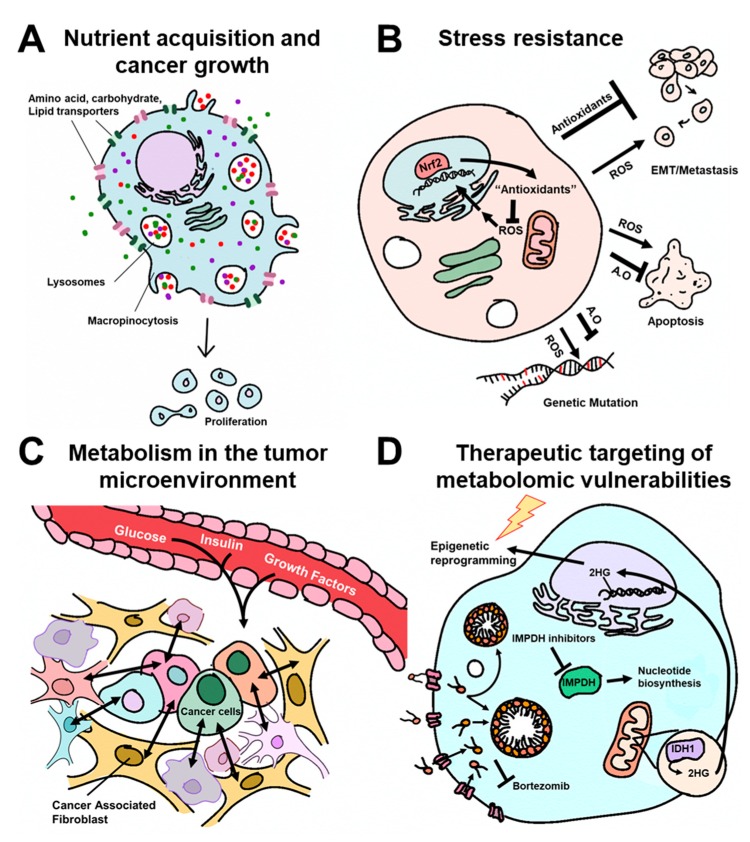
Schematic overview of the themes in the Special Issue on Metabolic Reprogramming and Vulnerabilities in Cancer. A.O: Antioxidants. Figure design by Heather Giza.
